# The effect of a workstation chair and computer screen height adjustment on neck and upper back musculoskeletal pain and sitting comfort in office workers

**DOI:** 10.4102/sajp.v71i1.279

**Published:** 2015-11-10

**Authors:** Nicole van Vledder, Quinette Louw

**Affiliations:** 1 Nicole van Vledder Physiotherapy, Diep River, South Africa; 2 Division of Physiotherapy, University of Stellenbosch, South Africa

## Abstract

**Aims:**

To assess the effect of a vertical height adjustment of the chair and visual display unit (VDU) on work-related upper quadrant musculoskeletal pain (WRUQMP) and sitting comfort in office workers. The upper quadrant refers to the occiput, cervical and upper thoracic spine, including the clavicles and scapulae.

**Methods:**

A single subject (*N* = 1) study, in which the subject serves as her own control, was conducted using the ABC design. An ergonomic workstation adjustment of VDU and chair height was compared to the subject’s usual workstation settings. Pain and sitting comfort was measured using visual analogue scales (VAS). The subject was assessed over three four-week phases as she performed her typical VDU work. The results were compiled and tabulated.

**Results:**

Both the mean and variance in pain intensity decreased after the workstation intervention. A deterioration in sitting comfort was noted.

**Conclusion:**

The vertical height adjustment of the chair and visual display unit may have contributed to a decrease in work-related upper quadrant musculoskeletal pain in this subject. This safe, economical workstation intervention may be a practical management option for the computer user suffering from work-related upper quadrant musculoskeletal pain. Further research into the measurement of comfort whilst sitting at a computer workstation is recommended.

## Introduction

Prolonged computer use has become customary in present-day office work environments (Wahlstrom et al. 2004). An associated increase in work-related upper quadrant musculoskeletal pain (WRUQMP), especially amongst those who are intensive computer users is also evident (Cagnie et al. 2007; Jensen 2003; Pillastrini et al. 2010). The upper quadrant refers to the occiput, cervical and upper thoracic spine including the clavicles and scapulae (Brink & Louw 2013). This increase in WRUQMP in computer users is of individual as well as economic concern (Waersted, Hanvold & Veiersted 2010), with notable economic cost implications because of absenteeism, decreased productivity and health care expenditure (Heinrich, Blatter & Bongers 2004). The neck is one of the most susceptible areas for musculoskeletal disorders (MSD) in computer users (Cagnie et al. 2007; Jensen 2003) with prevalence rates of 65% – 75% reported (Cho, Hwang & Cherng 2012; Griffiths et al. 2012; Kaliniene et al. 2013; Tornqvist et al. 2009).

Of concern is that prevalence rates have not decreased over the past three decades, despite efforts to address this problem in the workplace. These workplace interventions are further complicated as a result of the multidimensional nature of the problem, with non–modifiable and modifiable risk factors applicable (Johnston et al. 2009). Non-modifiable risk factors include higher age (older than 30 years) (Cagnie et al. 2007), female gender (Evans & Patterson 2000; Paksaichol et al. 2012; Waersted et al. 2010) and a previous history of neck pain (Paksaichol et al. 2012). Modifiable risk factors include the physical office environment, psychosocial workplace factors and workstation postural factors (Aarås et al. 1998), the latter being the focus of this study. Furthermore, physical and psychosocial factors in the workplace have been shown to interact in their effect on neck pain (Johnston et al. 2009) with high supervisor support shown to buffer physical risk factors such as increased time spent on computer tasks and an incorrectly positioned visual display unit (Johnston et al. 2009).

Numerous studies have been undertaken to identify which factors inherent in the workstation layout are most associated with WRUQMP (Andersen et al. 2011). Prolonged sitting at ergonomically poor workstations has been associated with MSD (Aarås et al. 1998). The chair influences the position of the computer user in relation to his or her keyboard and VDU and, consequently, the musculoskeletal demands placed on the worker (Gerr, Marcus & Monteilh 2004). A correctly adjusted chair has been shown to significantly reduce neck pain in seated workers (Rempel et al. 2006) with a recent review demonstrating that chair interventions have the potential to reduce MSD amongst workers who are required to sit for prolonged periods (Van Niekerk, Louw & Hillier 2012) Similarly, VDU and keyboard height in relation to the computer user has been investigated (Gerr, Monteilh & Marcus 2006; Straker & Mekhora 2000). VDU height has been shown to affect neck alignment, with prolonged neck postures in which the neck is either bent forwards (flexed) or arched back (extended), associated with neck MSD in computer users (Cagnie et al. 2007; Kaliniene et al. 2013; Pillastrini et al. 2010; Psihogios et al. 2001; Straker & Mekhora 2000). Likewise, keyboard placements in which the keyboard is higher than elbow level, have been associated with increased stiffness in the upper trapezius muscle (Faucett & Rempel 1994). Also, keyboard placement at, or slightly below, elbow level, has been associated with a reduced risk of neck MSD (Gerr et al. 2006; Waersted et al. 2010). Thus, the body alignment required from the office worker, when working at an inadequately adjusted computer workstation, may contribute to an elevated risk of WRUQMP (Straker et al. 2008).

However, the causal relationship of the computer workstation posture and MSD has been questioned (Andersen et al. 2011; Boocock et al. 2007; Brewer et al. 2006). Reasons for this uncertainty include the mixed outcomes yielded by ergonomic intervention studies (Andersen et al. 2011) and multifaceted interventions which preclude distinct deductions relating to workstation adjustments and WRUQMP (Esmaeilzadeh, Ozcan & Capan 2014). Additionally, the incomplete control of known confounding factors, such as workplace psychosocial factors and ergonomic advice, has been a severe methodological problem in the literature (Gerr et al. 2006). A review by Leyshon et al. (2010) did report moderate evidence that ergonomic workstation redesign improves comfort; however, no single ergonomic intervention was strongly supported. Recent randomised controlled trials (RCTs) have reported a reduction in WRUQMP following chair and VDU height adjustments in the intervention groups only (Esmaeilzadeh et al. 2014; Levanon et al. 2012). However, these studies included multiple ergonomic changes such as ergonomic training, stretching exercises and minibreaks, making it difficult to determine the effect of the workstation adjustment alone. In contrast, a RCT conducted by Gerr et al. (2005), concluded that adjusting the workstation chair and VDU height, with additional wrist and mouse positional adjustments, was unlikely to reduce the risk of WRUQMP in computer users.

Clinical advice, workplace policies as well as government legislative policies need to be based on trustworthy scientific guidance (Waersted et al. 2010). However, a strong level of evidence is still not available to support the viability of specific ergonomic interventions in WRUQMP in computer users (Andersen et al. 2011). Further research is thus needed, with clearly defined results (Leyshon et al. 2010) and adequate control of confounding factors (Gerr et al. 2006), to be useful to professionals working directly with WRUQMP (Leyshon et al. 2010).

A simple vertical adjustment of only the chair and VDU height, without confounding advice or other treatment, has not been identified in the literature reviewed to date. This practical intervention would be economical and easy to implement, facilitating self-management for the office worker suffering from WRUQMP. Training time and resources add to the expense of an intervention and compliance with postural and ergonomic advice requires active participation from the computer user, who is often distracted by the workload. Therefore, an intervention which does not require any participation from the worker beyond an initial basic chair and/or VDU height adjustment is appealing.

This study was carried out to ascertain whether adjusting only the vertical height of the chair and VDU in relation to the computer user, would affect WRUQMP and sitting comfort. The hypothesis is that WRUQMP and sitting comfort would be reduced following this ergonomic intervention. The basis for this intervention is that a change in the worker-workstation interface alters the postural demand placed on the worker, and subsequently the demand on the musculoskeletal system.

## Methodology

### Study design

A single subject experimental series type ABC, with 4 weeks per phase, was conducted. It was hypothesised that an adjustment of the chair and VDU height would reduce the subject’s WRUQMP and improve sitting comfort.

### Subject description

Subjects were eligible if they were office workers who used a computer for at least 5 hours per day and experienced neck or upper back symptoms associated with computer use that had been persistent or recurrent over the past 3 months. Additionally, the workstation of eligible participants had a seat and/or VDU height that was not within 10% of the seat and VDU height recommended in the literature (Hochanadel 1995). Potential participants were excluded from the study if they had neurological or other pathology, or had previous cervical or upper thoracic surgery or trauma that may contribute to the neck and upper back pain.

Furthermore, potential participants were excluded if they were undergoing treatment for neck or upper back pain as this may modify their pain or comfort. Additionally, respondents who had a BMI score of greater than 30, were pregnant or smokers, or used bifocal glasses were excluded as these factors influence body anthropometry and/or musculoskeletal discomfort (Borg-Stein, Dugan & Gruber 2005; Brage & Bjerkedal 1996; Doll, Petersen & Stewart-Brown 2000). A screening questionnaire was used to identify eligible subjects.

## Ethical considerations

Approval for the study was obtained from the Committee of Human Research at the University of Stellenbosch. The participant signed informed consent.

## Study procedures

### Recruitment

The study population – office workers in the administration department of Constantiaberg Mediclinic – was selected because of its proximity to the researcher’s own workplace. A letter was sent to the human resources department at Constantiaberg Mediclinic requesting permission to conduct the study. Permission was granted on 04 September 2013. All the office workers in the administration department who were at work that week completed a screening questionnaire, which included the inclusion and exclusion criteria for the study, in order to identify eligible subjects.

### Study phases

During phase A, the baseline phase, no change was made to the workstation. Phase B was the intervention phase and the workstation (chair and VDU height) was then adjusted as shown in Table 1 (Hochanadel 1995). The desk height was chosen as the fixed reference point from which the chair height and VDU height adjustments were calculated (Hochanadel 1995).

**TABLE 1:** Recommended workstation measurements.

Measurement	Description of measurement
Elbow height	desk height to floor + 25 mm
Elbow to seat distance	olecranon (with the subjects’ upper arm relaxed at their side, and the elbow flexed to 90’) to the seat
Eye to seat distance	corner of the subject’s eye to the seat
Intervention seat height	‘elbow height’ – ‘elbow to seat’ distance
Intervention visual display unit height	‘seat height’ + ‘eye to seat’ distance
Foot rest	The participant already had an adequate footrest which she was encouraged to use once her chair height was altered to allow her feet to rest on a firm surface

*Source*: Hochanadel, C.D., 1995, ‘Computer workstation adjustment: A novel process and large sample study’, *Applied Ergonomics* 26(5), 315–326. PMID: 15677034, http://dx.doi.org/10.1016/0003-6870(95)00035-6

No further ergonomic intervention or education was offered and the subject continued with her usual work for 4 weeks. At the start of phase C, the subject was informed that she was now free to change her workstation parameters, should she choose to do so.

### Outcome measures and measurement time frames

**FIGURE 1:**
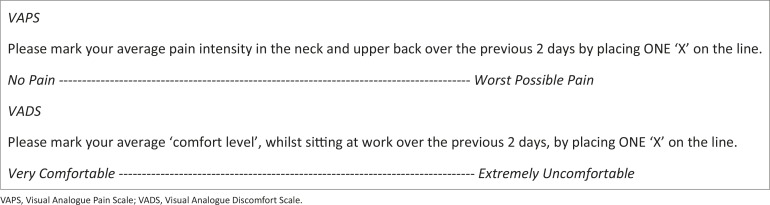
Visual Analogue Pain Scale and Visual Analogue Discomfort Scale. VAPS, Visual Analogue Pain Scale; VADS, Visual Analogue Discomfort Scale.

The primary outcome was neck and upper back pain intensity and the secondary outcome was comfort level whilst sitting at work. Each outcome was measured twice a week, at the end of the workday on a Tuesday and Thursday, with a Visual Analogue Pain Scale (VAPS) and Visual Analogue Discomfort Scale (VADS), as shown in Table 2 and Figure 1. The subject posted the completed forms into a sealed box which was provided by the researcher and kept at the subject’s workstation.

**TABLE 2:** Measurement time frames.

Study phase	Measurements per week	Measurements per phase
A	2 VAPS	8
	2 VADS	8
B	2 VAPS	8
	2 VADS	8
C	2 VAPS	8
	2 VADS	8

The VAS is a self-report instrument consisting of a 100 mm horizontal line, which the subject was asked to complete by making a mark on the relevant line to indicate her pain intensity and comfort during the previous 2 working days.

In comparison with discrete scales, measurement by a VAS is more exact, and the scale needs less explanation for the research participants (Reips & Funke 2008). Validity has been demonstrated with a correlation coefficient of 0.95 when compared to the McGill Pain Questionnaire and the Numeric Pain Scale (Ferraz et al. 1990). Test retest reliability was established at 0.71–0.99 (Ferraz et al. 1990). The researcher measured the distance from the ‘no pain’ and ‘very comfortable’ anchor labels to the point marked by the subject.

### Measurement of potential known confounding factors

Known confounding factors were monitored as follows at various stages of the study.

#### At entry to the study

The eligible subject was interviewed and examined by the researcher using a physiotherapy orthopaedic assessment (Petty 2011). This assessment provided information relating to the following: co-morbidities, psychosocial workplace factors, the nature of the job, frequency of breaks during the day, frequency of physical activity during the week, the physical work environment, mattress, pillow or wearing of prescription glasses, as well as an open question regarding any other factors the subject may presume to be related to her WRUQMP.

The 5 item Keele Generic Tool was included at the time of entry and exit from the study as psychosocial factors significantly affect pain intensity, and a change in psychosocial factors within the study period may therefore have introduced a confounding factor into the study. This is the psychosocial subscale of the STarT Back Tool, modified to screen and identify distress in conditions other than lower back pain. The Keele 5 item STarT generic screening tool was developed by Hill et al. (2010). The Chronbach’s alpha was 0.74 for this five psychosocial item subscale and substantial test-retest reliability (0.76) has been demonstrated in lower back pain (Hill et al. 2010). No study was found to use this tool specifically for neck and upper back pain.

#### At exit of the study

At the end of phase C the subject completed an Exit Questionnaire to assess any change in these known confounding factors, as mentioned above at time of entry to the study. This would allow the researcher to consider these factors when interpreting the data.

#### Twice weekly throughout the study

The subject indicated if she had taken any medication for her neck or upper back pain over the previous 2 working days, each time she completed the VAPS and VADS. This was necessary to establish whether the use of analgesia had affected the pain and comfort level reported.

#### At the end of each phase

The subject completed a questionnaire at the end of phases A and B, in which she reported the following: if she had received any treatment for her neck or upper back, altered the workstation herself or if there were any other factors over the past 4 weeks which may have influenced her work-related symptoms.

At the end of phase C, the Exit Questionnaire included these phase end questions.

A brief exit interview was carried out to assess the subject’s overall experience of the study, and specifically her understanding of the VADS. This exit interview was carried out in an attempt to understand the discrepancy in the subject’s verbal comments to the researcher, and her reporting on the VAPS and VADS.

### Data analysis

All data were captured on a Microsoft Excel 2010 spread sheet and descriptive statistics were used to describe the data set. As a measure of central tendency the mean was calculated and the range was calculated as a measure of variability for each phase, for the outcomes of pain and comfort. Measurements for A6 were not possible as the subject was absent that Thursday. The 2SD band method could not be used for the outcome of pain as the variance resulted in a negative -2SD value, which is not plausible for a VAPS as its lowest value is 0. The effect sizes for pain and comfort were calculated. Line graphs were drawn using Microsoft Excel 2010 to depict the trend for the outcome measures of pain and comfort.

## Results

### Study population and subject description

**FIGURE 2:**
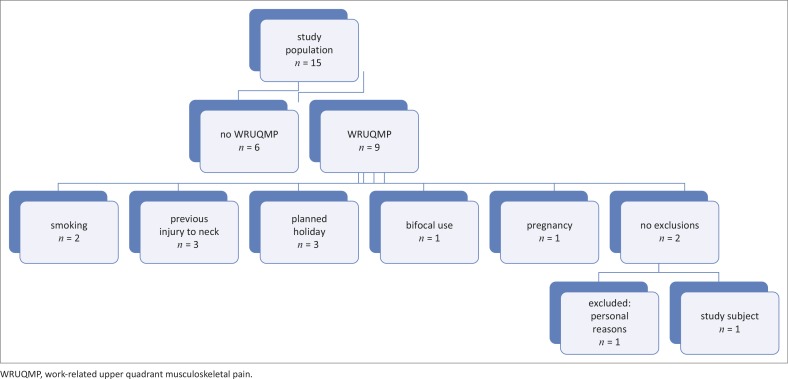
Flowchart of the recruitment process. WRUQMP, work-related upper quadrant musculoskeletal pain.

Fifteen office workers completed the screening questionnaire, with Figure 2 showing how the subject for the study was recruited. Some potential participants were excluded from the study because of more than one reason.

**FIGURE 3:**
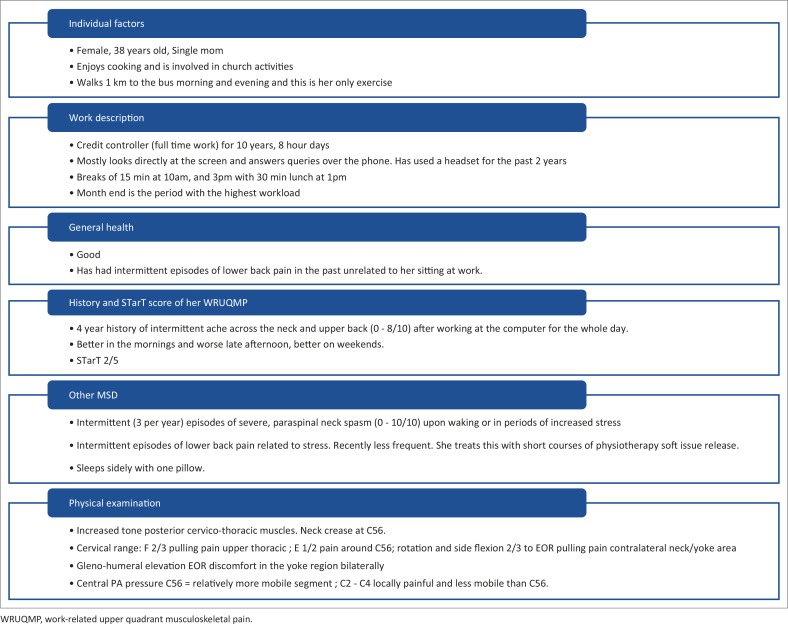
Subject interview and physical examination. WRUQMP, work-related upper quadrant musculoskeletal pain.

Figure 3 shows the interview and physical examination information gained for the study subject. Table 3 lists her baseline workstation measurements (phase A) and the measurements used to adjust the workstation for the intervention (phase B).

**TABLE 3:** Subject workstation measurements.

Workstation variable	Chair	VDU
Usual height	470 mm	1360 mm
Adjusted height	515 mm	1235 mm
Mismatch	45 mm = 9.6% (chair too low)	125 mm = 9.2% (VDU too high)

VDU, visual display unit.

**FIGURE 4:**
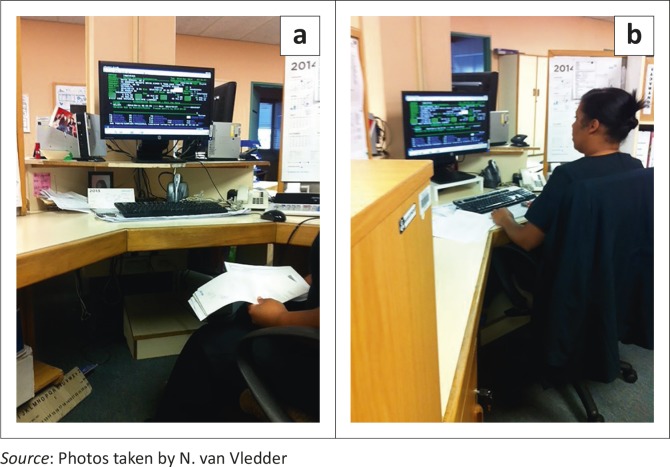
Photographs of the subject’s workstation: (a) Before workstation adjustment; (b) after workstation adjustment.

The photographs in Figure 4 show that although the chair and VDU height mismatches were between 9% and 10%, the difference in the pre- and post intervention VDU height was relatively greater. This was as a result of the pre-intervention height relationship in which the VDU was too high in addition to the chair position being too low.

The subject chose not to adjust her workstation during phase C, preferring to keep it at the phase B intervention adjustment heights.

### Outcome measures

#### Pain intensity

**FIGURE 5:**
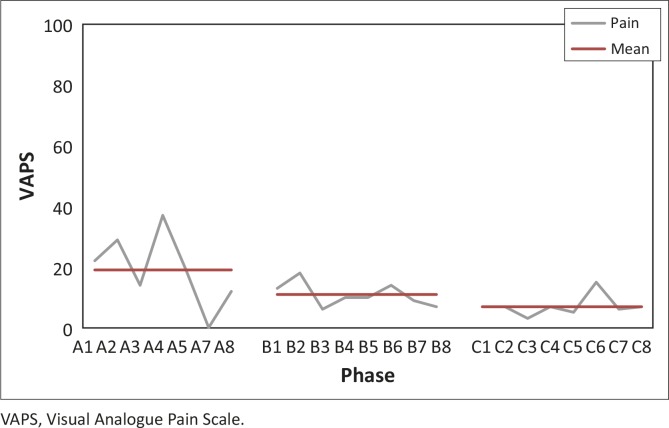
Visual Analogue Pain Scale measurements with the mean for each phase. VAPS, Visual Analogue Pain Scale.

Figure 5 shows the trend for pain intensity over the three study phases and the mean value for each phase. The mean pain level decreased from phase A to phase C. The effect size for pain intensity from phase A to phase B was 0.67 and the effect size from phase A to phase C was 1.0. This shows a small yet durable effect, which was maintained from phase B to phase C.

#### Comfort level whilst sitting at work

**FIGURE 6:**
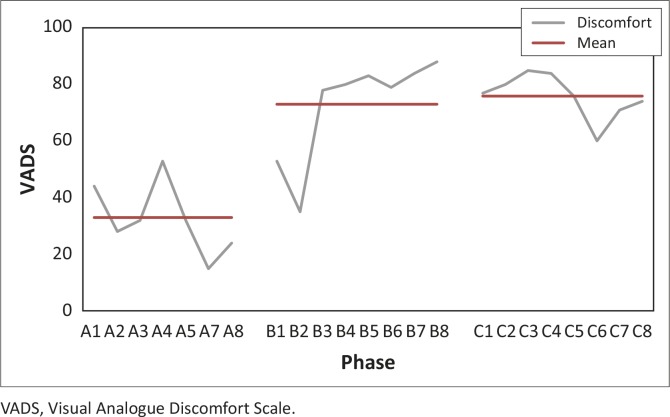
Visual Analogue Discomfort Scale measurements with the mean for each phase. VADS, Visual Analogue Discomfort Scale.

Figure 6 shows the trend for comfort level whilst sitting at work over the three study phases, and the mean value for each phase. Higher VADS scores were obtained in phases B and C, with corresponding higher mean values for discomfort in these phases. The effect size for comfort level whilst sitting at work from phase A to phase B was 3.17 and the effect size from phase A to phase C was 3.4. This shows a medium effect for an increase in discomfort which was maintained from phase B to phase C.

Table 4 shows the means and ranges for all phases for the outcomes of pain and comfort. The mean for pain reduced by 12 mm from phase A to phase C. The variability of pain as indicated by the range (minimum to maximum) also reduced from 37 in phase A to 12 in phase C. The trend for the data for comfort shows an increase in the mean discomfort scores of 43 mm from phase A to phase C. The variability of discomfort as indicated by the range (minimum to maximum) increases initially in phase B before decreasing in phase C.

**TABLE 4:** Means and ranges per phase for the outcomes of pain and comfort.

Outcome measure	Mean and range	Phase A	Phase B	Phase C
Pain Intensity	Mean (Min-Max)	19 (0–37)	11 (6–18)	7 (3–15)
	Range	37	12	12
Discomfort	Mean (Min-Max)	33 (15–53)	73 (35–88)	76 (60–85)
	Range	38	53	25

#### End of phase and end of study (exit) questionnaires

*End of phase*: Table 5 shows the known confounding factors assessed at the end of each study phase. These factors were constant over the 3 month study period.

**TABLE 5:** Assessment of known confounders (end of phase).

Confounding variable	Phase 1	Phase 2	Phase 3
Absent	Yes, 1 day	Yes, 1 day	No
Other treatment received	No	No	No
Own workstation adjustments	No	No	No
Open question: Anything else relevant	No	No	No
Pain medication used for neck or upper back	No	No	No

*End of study (exit)*: Table 6 shows the known confounding factors assessed at the time of exit of the study and compared to these factors at entry to the study. These factors were constant over the 3 month study period.

**TABLE 6:** Assessment of known confounders (end of phase C and relating to the previous 3 months).

Confounding variable	Subject’s response
Change in the nature of the work	No
Change in physical work environment	No
Change in exercise frequency	No
Change in family and social life	Grandfather died day after measurement C4
Accidents or injuries	No
Changes in general health	No
Change in mattress or pillow	No
Change in glasses prescription	No
STarT Generic Screening Tool	Study entry: 2/5 (low); Study exit: 0/5 (low)

*Exit interview*: The researcher briefly interviewed the subject regarding her overall experience of the study one week after completion of the study data collection. The subject verbally reported to be ‘much more comfortable’ after the intervention and was surprised that her VADS (Figure 1) scores had reflected that she was more uncomfortable. She indicated that she could have misunderstood the VADS. Furthermore, she reported that her intermittent familiar lower back muscle tightness had increased over the previous few days, but did not know of any specific reason for this increase.

## Discussion

WRUQMP is a common problem in office workers who use computers (Cagnie et al. 2007). Ergonomic intervention studies aimed at reducing WRUQMP have yielded mixed outcomes (Andersen et al. 2011). Uncertainty exists, therefore, amongst clinicians as to which workstation adjustments to recommend. The finding in this study suggests that a chair and computer screen height adjustment may reduce WRUQMP in computer users.

In our study, the trend for WRUQMP intensity decreased after the intervention was introduced (Figure 5). The subject was a 38 year old full-time female office worker, who uses a computer for most of her 8 hour work day. The subject’s mean pain level decreased by 12 mm during phase C compared to the baseline level in phase A. This decrease in pain is less than the 20 mm required to be a clinically important difference (CID) in chronic pain (Ostelo et al. 2008). Thus, although the mean pain decreased by half of the mean intensity of pain during the baseline phase, it is uncertain whether the change was meaningful to the subject. In the future, it is suggested that the patients’ perception of what would constitute a clinically meaningful change should be assessed before commencement of the study.

The variability of pain decreased during the intervention and last phases. The reduced variability from phase A indicated a positive effect of the intervention, as more stability in symptoms was noted during the latter two phases. As the pain did not increase during the period of increased workload at month end, it affirms the improvement in her symptoms. The ergonomic intervention may thus have had a buffering effect on the pain intensity during periods of increased workload. Our findings pertaining to pain intensity and variability of pain are consistent with ergonomic workstation intervention studies which have reported a decrease in WRUQMP (Esmaeilzadeh et al. 2014; Hochanadel 1995; Levanon et al. 2012; Mekhora et al. 2000).

Esmaeilzadeh et al. (2014) also investigated subjects who experienced WRUQMP and used a VAPS for the outcome measure of pain intensity. The subjects in their study were requested to report symptoms during the previous 3 months. Our outcome measures were assessed more frequently with a symptom recall period of only 2 days, possibly enabling more accurate symptom report. Furthermore, the study by Esmaeilzadeh et al. (2014) included comprehensive ergonomic training as well as workstation adjustment. This training consisted of two theoretical and practical interactive ergonomic lessons, each 90 minutes long, conducted by the investigators who were qualified in ergonomic training. In addition, participants in the intervention group received an ergonomic training brochure which consisted of information about office ergonomics such as risk factors for WRUQMP, importance of prevention, workstation adjustments, and workplace exercises. Participants were taught how to adjust their individual workstations, and checked and encouraged to do so at monthly intervals. Similarly Levanon et al. (2012) included a comprehensive individual worksite adjustment (up to 6 weekly sessions with all equipment adjusted relevant to the worker’s anthropometrics), corrective exercise (for specific MSDs, muscle relaxation, including a home programme to be repeated twice daily) and minibreaks (brief muscle relaxation at the workstation and breaks of minutes accompanied by a computer announcement). Only the intervention groups reported a reduction in WRUQMP scores. In the studies by Esmaeilzadeh et al. (2014) and Levanon et al. (2012), the combination ergonomic intervention is suggested to reduce the WRUQMP, not the effect of the workstation height adjustment alone. However, the combined intervention does not enable the researchers to discern which aspect of the intervention was associated with the decreased pain reported. Hence, our study only focused on vertical adjustment to ascertain whether it can be used as a feasible and cost effective method to address WRUQMP.

Conversely to our findings, Gerr et al. (2005) showed that an ergonomic workstation intervention, similar to ours, was unlikely to reduce WRUQMP in computer users. This contradictory finding may be explained by two factors. Firstly, Gerr *et al*. reported that the relevant workstation adjustment was not always possible. This was primarily because of the required elbow position being impossible to achieve with the participant’s workstation. Hence, not all subjects in their study could potentially benefit from the workstation adjustment. Secondly, Gerr *et al*. reported that compliance, measured at the time of intervention and at two subsequent follow-up visits, was poor in their sample. In our study, compliance was good as the subject did not alter her workstation after the intervention phase. This difference in compliance may thus explain the difference in findings between our study and Gerr et al. (2005).

The subject in our study experienced low intensity pain, albeit frequent. This is typical of WRUQMP associated with computer use (Paksaichol et al. 2012; Punnett & Bergqvist 1997). The symptoms may be related to the subject’s workstation (Straker et al. 2008) as her VDU was too high for her anthropometry (Table 3 and Figure  4). Thus, she had to look up at the screen, resulting in a ‘thrown back’ head position, hinging on the mid-lower cervical spinal structures.

This neck position has previously been significantly associated with neck MSD in computer users (Kaliniene et al. 2013), possibly as a result of increased cervical spine compressive loading of the posterior spinal structures and a creep response in the soft tissues (Edmondston et al. 2011; Harms-Ringdahl et al. 1986). In addition her elbows were below the level of the desk as her chair was too low (Table 3 and Figure  4). This relatively higher keyboard position has been shown to be associated with neck MSD in computer users (Gerr et al. 2006; Waersted et al. 2010). This position demands sustained shoulder blade elevation to reach the keyboard, further increasing posterior cervico-thoracic and upper trapezius muscle activity (Faucett & Rempel 1994; Straker & Mekhora 2000). It has been hypothesised that the physiological consequences of this muscle overuse may result in localised muscle fatigue (Visser & Van Dieën 2006), with insufficient muscle relaxation of low threshold motor units (Hermens & Hutten 2002). This mechanism is thought to contribute to myofascial pain in computer users (Hagberg 1995). A workstation layout which enables a more neutral body alignment may result in less WRUQMP, because of reduced cervico-thoracic muscle activation (McLean 2005) and reduced strain on cervical structures. This may have been the mechanism for the reduction in pain intensity seen in our study.

Although a reduction in pain intensity was noted in our study, some WRUQMP remained. This is consistent with the findings by Hoyle et al. (2011) in which trapezius load was measured whilst doing computer typing work under three workstation postural stress conditions. In this study increased trapezius load and WRUQMP was noted after all three working conditions, even in conditions compliant with current ergonomic guidelines for office work. Hoyle et al. (2011) concluded that modification of the physical layout alone may not prevent musculoskeletal symptoms from occurring. Furthermore, Huysmans et al. (2012) have reported previous neck and upper back symptoms as being the most important risk factor for future symptoms amongst office workers. Potentially, increased tissue vulnerability and sensitisation of the pain system would explain the increased risk in this group. Our study subject had a previous history of WRUQMP and this may also be a further reason why she continued to experience some residual pain symptoms.

The secondary outcome of this study, to ascertain the effect of a chair and VDU height adjustment on comfort level whilst sitting at work, shows that the subject became more uncomfortable after the intervention phase (Figure 6). During the exit interview, the subject verbally reported to the researcher that she was comfortable at the workstation after the intervention. The subject indicated that the anchor labels ‘very comfortable’ and ‘extremely uncomfortable’ (Figure 1) may have caused confusion, with ‘greater comfort’ assumed to involve a mark further to the right on the VADS. Mekhora et al. (2000:367–379) reported using a VADS with anchors of ‘discomfort’ throughout (‘no discomfort’ vs ‘extreme discomfort’); however, when the VADS figure in the article is consulted the anchors are marked ‘no pain’ and ‘extreme pain’. Gerr et al. (2005:478–487) used a VADS in which subjects were asked to rate the ‘worst discomfort such as pain, aching, burning, numbness or tingling during the previous week’. Comfort is arguably a less often investigated construct compared to outcomes such as pain. Further research may be required to establish standardised methods to measure comfort. This will facilitate comparison between studies in the future.

Comfort has been defined as a ‘pleasant state or relaxed feeling of a human in reaction to its environment’ (Vink & Hallbeck 2012:271). Thus, a change in neck and upper back pain intensity needs not correlate to a change in perceived sitting comfort, as the concept of comfort includes other body parts in which pain is felt as well as environmental and psychosocial factors (Vink & Hallbeck 2012). Interestingly, the subject did suffer an episode of her familiar lower back muscle tightness one week after the end of the study period. It is possible that the decreased comfort level may have been an early indicator of this MSD (Lindegard et al. 2012; Wahlstrom et al. 2004), and that this may offer an alternative explanation for the increase in her VADS measurements.

WRUQMP in computer users is a multidimensional problem and various risk factors may interact to increase or buffer symptoms (Johnston et al. 2009). Physical, environmental and psychosocial workplace factors are acknowledged factors which may affect the experience of WRUQMP (Johnston et al. 2009). Thus, our study assessed potential confounding risk factors by means of questionnaires at entry, phase end and exit of the study and found known confounders to be constant (Tables 5 and 6). Published studies have also used questionnaires to control for confounding factors (Aarås et al. 1998; Mekhora et al. 2000). Monitoring known confounders enabled us to ascertain if any of these factors influenced the study outcomes. The findings illustrated that none of these confounding factors influenced the outcomes of this subject. This strengthens the validity of our findings because of the intervention.

The subject was not blinded to the intervention and, therefore, the placebo effect may result in bias if she is under the impression that superior workstation ergonomics have been implemented (Mekhora et al. 2000). Furthermore, the subject may have altered her behaviour because she was being observed as described by the Hawthorne effect (Adair 1984). In this case, the VAPS measurements would be expected to drop immediately at intervention and increase again gradually as measurements continued for 8 weeks after the workstation adjustment. This was not the case and the placebo and Hawthorne effects are therefore unlikely to have had a notable effect on study outcomes.

### Limitations and recommendations

This was a single subject study of a single intervention, and the result may not be generalised to other population groups and ergonomic interventions. Similar studies with greater numbers of subjects, or the combination of multiple single subject studies similar to the present study, would enhance the validity of the findings, therefore increasing the confidence with which clinicians may recommend this intervention. Furthermore, a desktop computer was used and the results of this study cannot be generalised to laptop, tablet or multiple screen workstation scenarios.

A strength of the studies by Esmaeilzadeh et al. (2014) and Levanon et al. (2012) is that these studies included self-report and objective workstation posture assessments respectively, at entry and exit of the study. Both studies reported an improvement in workstation posture. Esmaeilzadeh et al. (2014) viewed the use of self-report posture assessment to be a limitation of their study as it may introduce bias, and noted that an objective measure would be preferable. An objective account of sitting posture is a ‘superior method of postural examination compared to subjective or self-report measures as it can provide information about the biomechanical alignment of the bony structures at any specific moment in time’ (Brink & Louw 2013:281–288). No assessment of workstation posture and subsequent change to workstation posture after the workstation adjustment was included in our study and this is a limitation of the study.

Returning the subject’s workstation to baseline settings after phase B for a wash out period would have increased the validity of the findings. However, as this subject reported less pain after the intervention phase and chose to keep her workstation at the adjusted height, this may have been regarded as unethical. Information regarding the use of pain medication was only gained in relation to neck and upper back pain which may have introduced bias as pain medication for other areas would also have affected the WRUQMP.

## Conclusion

The aim of this study was to ascertain whether a chair and VDU height adjustment would reduce WRUQMP in office workers who are computer users. The findings of this single subject study suggested that the vertical height adjustment of the chair and VDU may have contributed to a decrease in WRUQMP in this subject. This safe, economical workstation intervention may be a practical management option for the computer user suffering from WRUQMP. However, the reduction in reported pain levels was too small to be considered clinically significant, and a deterioration in sitting comfort was noted. Further research with larger population studies and longer follow–up time frames is now required to affirm these findings in a representative sample.
